# Setup of an *In Vitro* Test System for Basic Studies on Biofilm Behavior of Mixed-Species Cultures with Dental and Periodontal Pathogens

**DOI:** 10.1371/journal.pone.0013135

**Published:** 2010-10-01

**Authors:** Kerstin Standar, Bernd Kreikemeyer, Sylvio Redanz, Wanja L. Münter, Michael Laue, Andreas Podbielski

**Affiliations:** 1 Institute of Medical Microbiology, Virology and Hygiene, University Hospital Rostock, Rostock, Germany; 2 Electron Microscopic Centre, Institute of Pathology, University Hospital Rostock, Rostock, Germany; Cinvestav, Mexico

## Abstract

**Background:**

Caries and periodontitis are important human diseases associated with formation of multi-species biofilms. The involved bacteria are intensively studied to understand the molecular basis of the interactions in such biofilms. This study established a basic *in vitro* single and mixed-species culture model for oral bacteria combining three complimentary methods. The setup allows a rapid screening for effects in the mutual species interaction. Furthermore, it is easy to handle, inexpensive, and reproducible.

**Methods:**

*Streptococcus mitis*, *S. salivarius* and *S. sanguinis*, typical inhabitants of the healthy oral cavity, *S. mutans* as main carriogenic species, and *Porphyromonas gingivalis*, *Fusobacterium nucleatum*, *Parvimonas micra*, *S. intermedius* and *Aggregatibacter actinomycetemcomitans* as periodontitis-associated bacteria, were investigated for their biofilm forming ability. Different liquid growth media were evaluated. Safranin-staining allowed monitoring of biofilm formation under the chosen conditions. Viable counts and microscopy permitted investigation of biofilm behavior in mixed-species and transwell setups.

**Findings:**

*S. mitis*, *F. nucleatum*, *P. gingivalis* and *P. micra* failed to form biofilm structures. *S. mutans*, *S. sanguinis, S. intermedius* and *S. salivarius* established abundant biofilm masses in CDM/sucrose. *A. actinomycetemcomitans* formed patchy monolayers. For in depth analysis *S. mitis*, *S. mutans* and *A. actinomycetemcomitans* were chosen, because i) they are representatives of the physiological-, cariogenic and periodontitis-associated bacterial flora, respectively and ii) their difference in their biofilm forming ability. Microscopic analysis confirmed the results of safranin staining. Investigation of two species combinations of *S. mitis* with either *S. mutans* or *A. actinomycetemcomitans* revealed bacterial interactions influencing biofilm mass, biofilm structure and cell viability.

**Conclusions:**

This setup shows safranin staining, microscopic analysis and viable counts together are crucial for basic examination and evaluation of biofilms. Our experiment generated meaningful results, exemplified by the noted *S. mitis* influence, and allows a fast decision about the most important bacterial interactions which should be investigated in depth.

## Introduction

Caries and Periodontitis are extremely frequent human diseases with high socioeconomic impact. They are associated with several potentially severe complications due to bacterial invasion of neighbouring anatomical structures or haematogenous spreading and purulent infections at distant sites. If not managed by appropriate therapies, both diseases are chronically progressive. Their pathogenesis is explained by a locally disturbed microecology within the bacterial biofilms covering the surfaces of the teeth and the subgingival sulci.

Bacterial biofilms are able to form and spread on the surfaces of the teeth in healthy oral cavities. Such biofilms display typical structural features such as i) a chemically conditioned support, ii) pioneer bacteria firmly adhering to the support's surface, iii) microcolony formation and production of macromolecular extracellular substances, iv) attachment of secondary colonizer binding to the growing biofilm, v) a predefined maximum thickness due to a balance between biofilm production and detachment (i.e. maturation) processes. These biofilms may contain up to several hundred bacterial species. These bacterial consortia are inconsistent between individual sites in one oral cavity and even more between diverse oral cavities [1]–[4].

Because of the extensive species variation between human individuals the concept of specific indicator bacteria for physiological and pathological biofilms in oral cavities is currently modified [5]–[9]. However, it is generally accepted that the cell number of the involved bacteria changes in a species-dependent manner during disease development. Simultaneously, individually differing species disappear below detection level while new species are temporally or constantly detectable during disease development [10], [11].

The involvement of many species and their constant qualitative and quantitative variations makes it extremely complicated to setup *in vitro* biofilms that truly reflect the natural situation. However, establishing of *in vitro* biofilms is still necessary to investigate substances suitable for the suppression of caries or periodontitis.

While the epidemiology of the microflora in healthy and diseased oral cavities has greatly been promoted by the introduction of advanced microscopic and molecular techniques, *in vitro* experiments have to rely on classical culture methods. It is currently impossible to grow representative *in vitro* multi-species biofilms resembling those encountered during caries or periodontitis. However, mixing a few important species to mimic biofilms encountered during complete health, transition to disease, developing disease, and finally, in deep lesions appears to be feasible. Such studies were performed in many laboratories [e.g. 12]–[20]. Depending on the scientific question in these laboratories different experimental setups were developed. Disparate setups and methods predominantly comprised 1) the species used in the studies, 2) the incubation conditions (static or flow, aerobic or anaerobic), which are very important and should mimic the environmental and physical parameters of the *in vivo* biofilm niche, 3) the documentation of biofilm formation, biofilm mass and biofilm maturation, and 4) the quantification of the individual species contained in the biofilms. Thus, a setup which could be used in many labs and which proves useful for many different scientific questions would be beneficial to basically investigate and understand biofilm formation and bacterial interaction in biofilm structures.

In the present study, we intended to set up conditions for the investigation of bacterial biofilm formation and combined three complimentary methods (cfu, safranine staining, microscopy) as a basis for multi-species culture investigation. We used *Streptococcus mitis*, *S. salivarius*, *S. mutans, S. sanguinis, S. intermedius, Aggregatibacter actinomycetemcomitans, Porphyromonas gingivalis, Fusobacterium nucleatum and Parvimonas micra* as bacteria associated with a healthy oral cavity, caries, and periodontitis, respectively. Based on the experimental protocols used in this study, we could demonstrate in mixed species assays as well as in assays employing separating filter membranes between the partners, increased or decreased contribution of single species to biofilm formation and effects on viability exclusively in one co-incubation partner.

## Results

### Evaluation of basic parameters of biofilm formation

Investigation of biofilm structures and bacterial interaction required establishment of reliable biofilm setup protocols. For this purpose, different culture media were tested in a static biofilm setup to evaluate the best conditions for *in vitro* simulation of biofilm generation. It is assumed that not much liquid exchange occurs during periodontitis *in vivo*, thus static conditions best mimic this situation and also allow the action of potential signalling molecules in mixed species cultures. Six different media were examined for their effect on mono-species biofilm formation for a time period up to five days. Safranin staining was employed as an easy read-out approach. This method is used for the determination of biofilm mass, comprising bacterial cells and extrapolymeric substances. Typically, an OD_492_ nm with a value of more than 0.05 is required to indicate biofilm formation. Lower values are mostly caused by scattered bacteria in monolayers (data not shown). For comparison, also the growth curves of planktonic cells were recorded for each culture medium. The table S1 summarizes the results for all bacteria analyzed. However, the present study will only focus on the detailed results of *S. mitis*, *S. mutans* and *A. actinomycetemcomitans* as representatives of the physiological, cariogenic and periodontitis-associated oral microflora, respectively.

**Figure 1:**
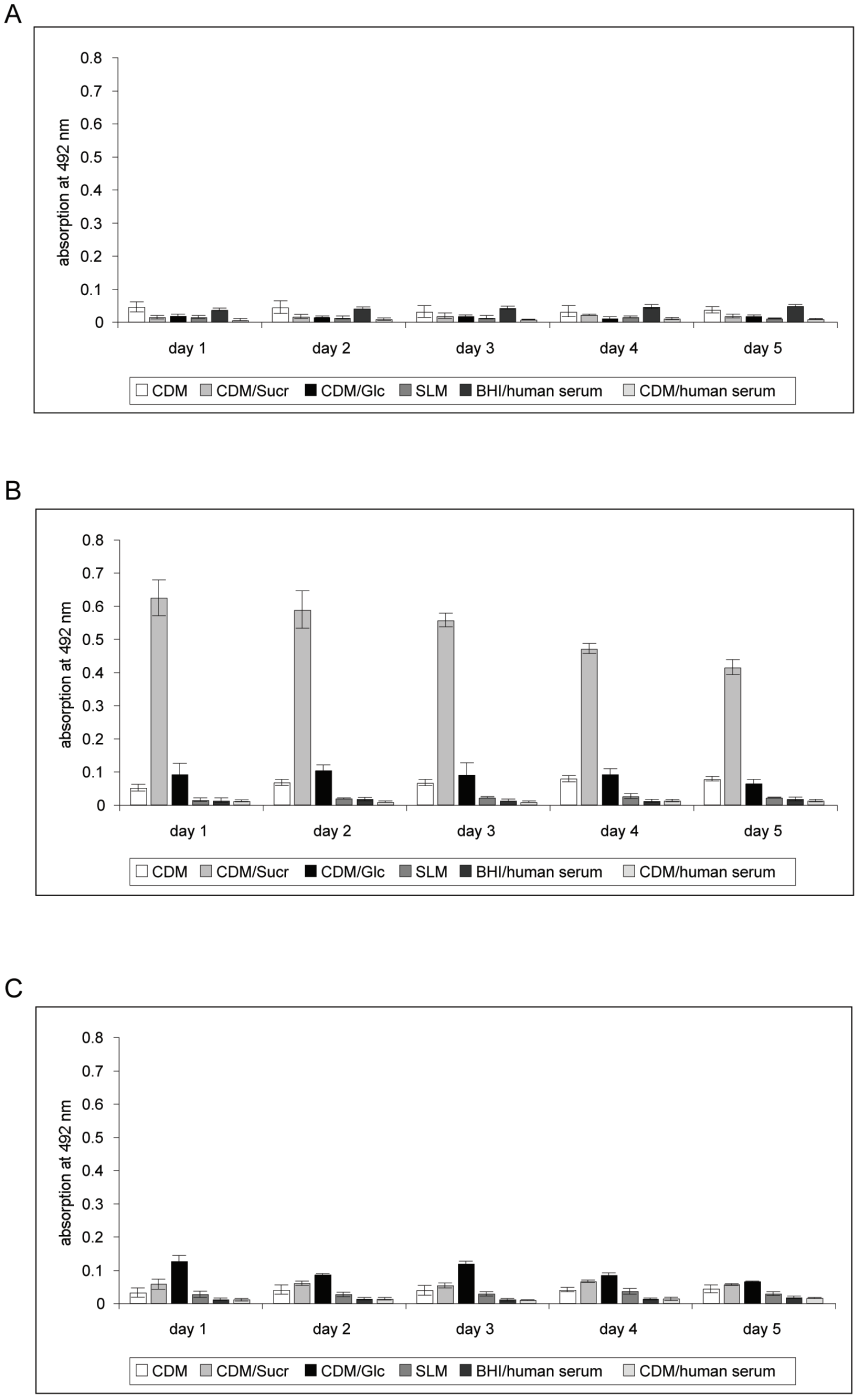
Safranin-staining assay of mono-species-cultures in different media. A), B), C) Results for *S. mitis, S. mutans. A. actinomycetemcomitans,* respectively. CDM - chemically defined medium, Sucr – sucrose, Glc – glucose, BHI – brain heart infusion, SLM- saliva-like medium.

Monitoring the *S. mitis*, *S. mutans* and *A. actinomycetemcomitans* mono-species cultures for biofilm mass over a period of five days showed that biofilm formation of *S. mutans* occurred within the first 24 hours of incubation time in CDM without glucose (chemically defined medium; for details of composition please refer to reference 54), CDM supplemented with 50 mM glucose (CDM/glc) or sucrose (CDM/suc). The highest amounts of biofilm cell mass was formed in CDM/sucrose. The biofilm mass reached at this time point remained stable during the residual observation period. For *A. actinomycetemcomitans*, scattered patches of monolayers appeared after one day of incubation in CDM/glucose or CDM/sucrose. No multi-layered structures were observed in any tested medium. *S. mitis* bacteria failed to establish biofilms in all tested media (figure 1).

Growth curves in CDM/sucrose revealed that *S. mitis* and *S. mutans* increased in their optical density. A parallel decrease in the medium pH within 24 hours was noted (7.7 to 4.77 and 5.8, respectively). Planktonic *A. actinomycetemcomitans* did not grow in this medium (figure S1). Nevertheless, determination of cfu/ml showed constant numbers of viable cells and a slight decrease of medium pH (7.7 to 7.47 within 24 h).

Next to the determination of biofilm mass and planktonic growth, viable cell counts and fluorescence microscopy were performed on the samples as outlined in the Materials/Methods section. In CDM/sucrose, the number of surface-adherent viable cells as measured by colony forming units per ml suspension (cfu/ml) decreased after day one of investigation for all three bacterial species. However, viable *S. mutans* and *A. actinomycetemcomitans* cells were retrievable at day five of incubation (table S2).

Thus, as the only medium supporting growth or at least viability of all three species while simultaneously allowing monolayer/biofilm growth for at least two species, CDM/sucrose was used for all subsequent experiments.

In this medium, biofilm formation of single bacterial species was also tested in fibronectin-coated wells. Although the biofilm mass was slightly different compared to uncoated plastic supports, all three species behaved similar concerning their biofilm forming ability or formation of monolayers, respectively (figure S2). Subsequently, the experiments were performed using uncoated supports.

### Biofilm behavior of two-species cultures

To approach the natural situation and to obtain information about the species interactions, in the next step we employed co-cultivation of *S. mitis* with *S. mutans* and/or *A. actinomycetemcomitans*.

**Figure 2:**
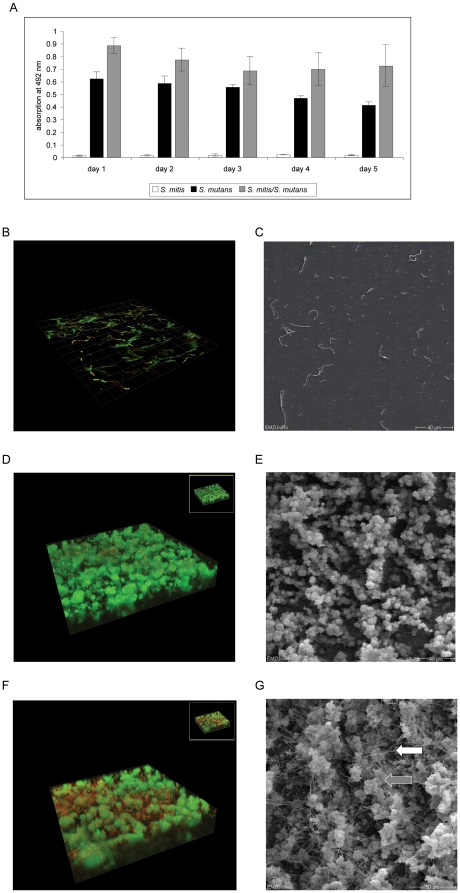
Safranin-staining assay, Confocal laser scanning microscopy (CLSM) and Scanning electron microscopy (SEM) of *S. mutans* mono-species biofilms, *S. mitis* adherent cells and *S. mutans*/*S. mitis* two-species biofilms. A) Safranin-staining assay of the mono- and two-species-biofilms of *S. mitis* and *S. mutans*. B) CLSM and C) SEM pictures of the *S. mitis* (mono-species) adherent cells. D) CLSM and E) SEM pictures of the *S. mutans* mono-species biofilm, F) CLSM and G) SEM pictures of the two-species combination *S. mitis*/*S. mutans*. For CLSM detection, cells were stained with Live/Dead dyes. Live cells are stained in green, dead cells light up in red. Grey arrow: *S. mutans* extracellular matrix structure; white arrow: *S. mitis* chain.

Safranin-assays revealed that the co-cultivation of *S. mutans* with *S. mitis* resulted in an increase of total biofilm mass compared to the *S. mutans* mono-species cultures (figure 2 A). Confocal laser scanning microscopy after live/dead-stain confirmed that *S. mitis* failed to form biofilms. In the two-species setting with *S. mutans* the integration of *S. mitis* within the biofilm structures was noted. Scanning electron microscopy confirmed this result (figure 2 B–F). Here, *S. mitis* chains could be found on and between the typical extracellular matrix structures synthesized by *S. mutans* (figure 2 E, grey arrow *S. mutans* extracellular matrix structure, white arrow *S. mitis* chain). Of note, within these mixed communities no colony forming units of *S. mitis* were detectable, whereas *S. mutans* numbers and viability were unchanged compared to single species settings (table S2). While incubating the two species, the culture fluid acidity reached pH 4.96 after 24 hours and decreased to pH 4.52 after 5 days.

**Figure 3:**
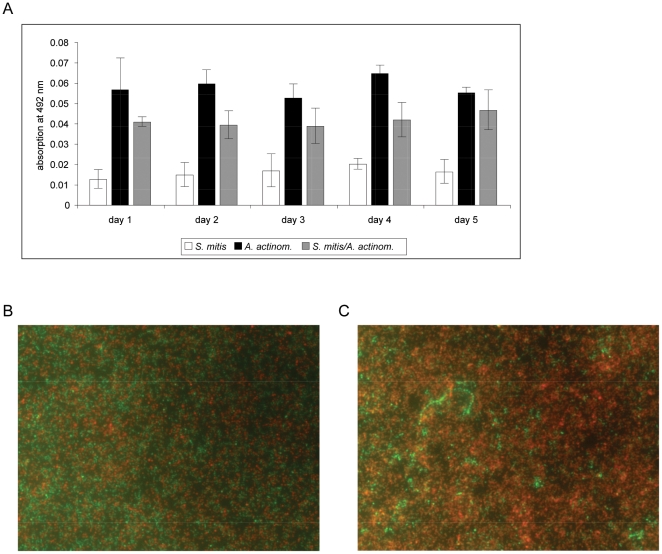
Safranin-staining assay and fluorescence microscopy of the *A. actinomycetemcomitans/S. mitis* two-species biofilms. A) Result of Safranin-staining assay of the mono- and two-species biofilms. B) Fluorescence microscopy of *A. actinomycetemcomitans* and C) *A. actinomycetemcomitans*/*S. mitis* biofilms. For the assay the cells were stained with the Live/Dead dyes. Live cells are stained in green, dead cells light up in red. Magnification: 400×.

Combining *S. mitis* with *A. actinomycetemcomitans* resulted in safranin-stain values that were lower compared to the mono-species culture of *A. actinomycetemcomitans* (figure 3 A). Concomitantly, viable cell counts decreased for both *S. mitis* and *A. actinomycetemcomitans* as compared to single species incubations under the chosen conditions (table S2). Furthermore, the culture fluid pH reached values around 4.55 at day 1, which were lower than those of *S. mitis* and *A. actinomycetemcomitans* mono-species cultures (pH 4.77 and 7.47, respectively, day 1).

SEM and fluorescence microscopy revealed that the surface of the wells was mainly covered by *A. actinomycetemcomitans* cells with few *S. mitis* cells on top of their partner cells. Finally, as determined by live/dead stain and viable counts, cells of *A. actinomycetemcomitans* died faster in the presence of *S. mitis* compared to mono-species cultures (figure 3 B and C, and table S2, respectively).

### Successive seeding strategy

The results from simultaneously seeded two-species cultures indicated the presence of bacterial interaction mechanisms. The aim of the next set of experiments was to evaluate the influence of timing of bacterial adherence on biofilm formation. Thus, we next employed a successive seeding strategy.

**Figure 4:**
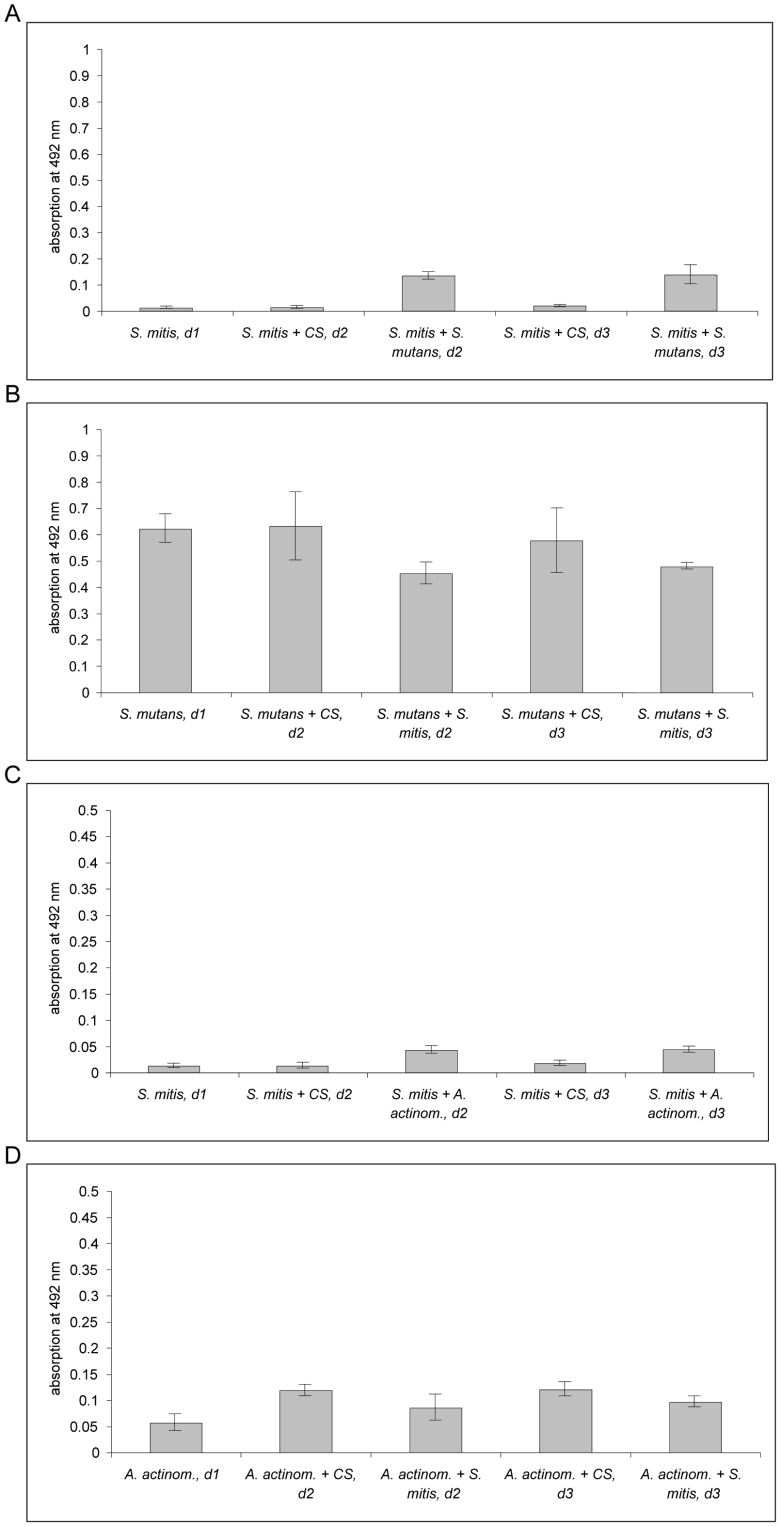
Successive seeding of *S. mitis* and *S. mutans* or *A. actinomycetemcomitans* combinations in biofilm experiments and vice versa. In both experiments, on day 0 *S. mitis, S. mutans or A. actinomycetemcomitans* was inoculated into separate wells. Following incubation of 24 hours, biofilm mass of the mono-species was determined by safranin-stain (d1 =  day 1). In parallel, *S. mutans* or *A. actinomycetemcomitans* was inoculated to *S. mitis* or, in reverse order, *S. mitis* to *S. mutans* or *A. actinomycetemcomitans*. After further incubation for 24 or 48 hours, biofilm mass was again quantified by safranin-staining (d2 =  day 2 and d3 =  day 3). The graph shows the data obtained by safranin-staining for the combination *S. mitis* with *S. mutans* and vice versa (A and B) and *S. mitis* with *A. actinomycetemcomitans* and vice versa (C and D). For better optical discrimination, the grading of the y-axis is different in both graphs. CS – chemically defined medium with sucrose.

The data obtained from the safranin-assays showed that inoculation of *S. mutans* on top of *S. mitis* lead to biofilm formation (figure 4 A). However, biofilm mass was not as abundant as for the *S. mutans* mono-species culture (compare figure 2 A). In the reverse setup, when *S. mutans* was used as the primary colonizer, the results showed a decrease of biofilm mass compared to the *S. mutans* mono-species culture as well as after seeding with CDM/sucrose as control (*S. mutans* + CS, d2 and d3; figure 4 B). Nevertheless, in both cases the number of *S. mutans* colony forming units did not differ from those of simultaneously seeded cultures (table S3). For *S. mitis* no colony forming units were detectable in such successive seeding combinations.

**Figure 5:**
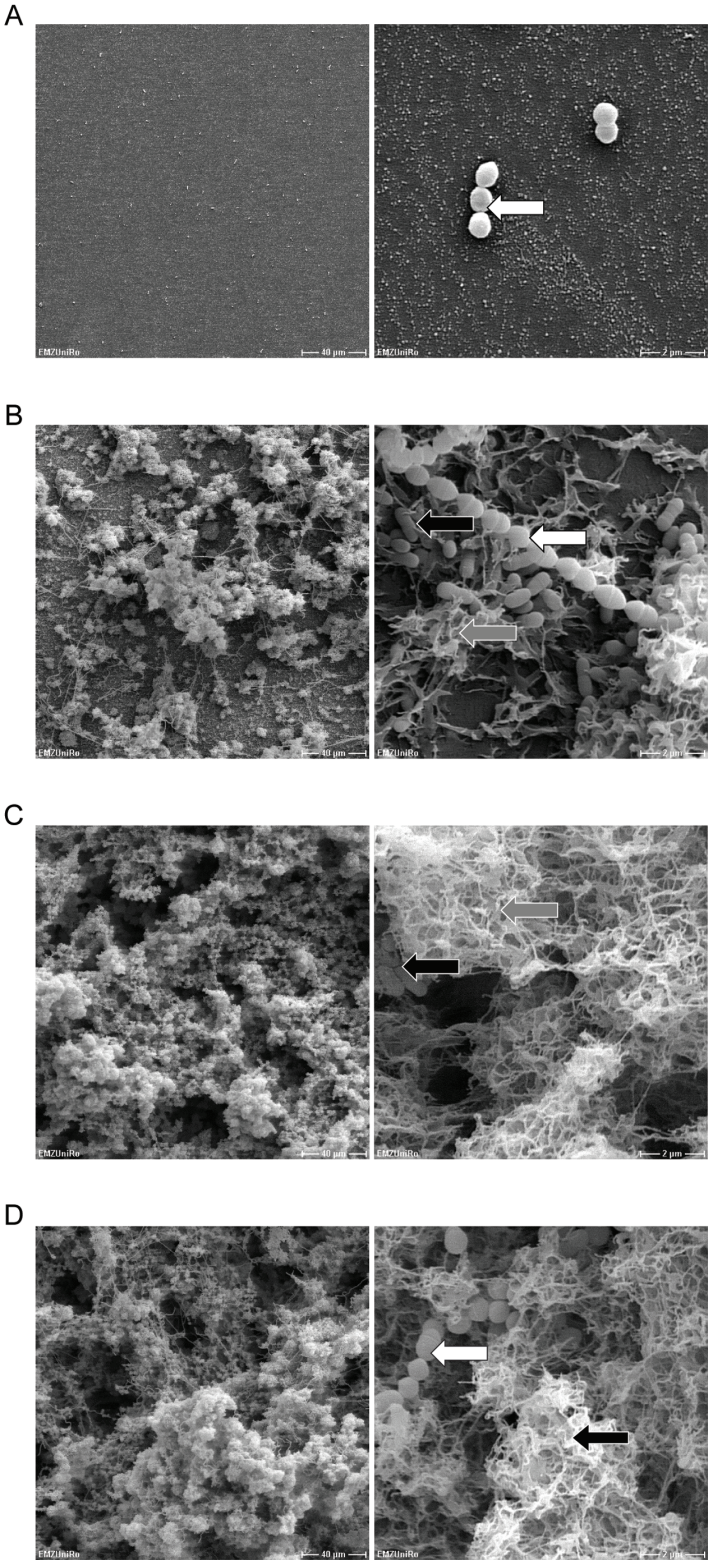
SEM analysis of biofilms obtained by successive seeding of *S. mitis* and *S. mutans*. A) *S. mitis* mono-culture. B) *S. mitis* as first colonizer, *S. mutans* as second species. C) *S. mutans* mono-culture. D) *S. mutans* as first colonizer, *S. mitis* inoculated as second species. Magnification 500× and 10 000×. White arrows: *S. mitis*; Grey arrows: *S. mutans* extracellular matrix structure; Black arrows: *S. mutans.*

In order to visually complement the results from the *S. mitis*/*S. mutans* successive seeding assays we performed scanning electron microscopy (SEM). As shown in figure 5 A–D, SEM pictures were consistent with the data obtained from safranin-stain. The inoculation of *S. mutans* to *S. mitis* led to biofilm formation, dominated by *S. mutans* and its extracellular matrix structures (figure 5 B, black arrow *S. mutans*, white arrow *S. mitis*, grey arrow *S. mutans* extracellular matrix structure). In the reverse order, the inoculation of *S. mitis* to *S. mutans* showed again the integration of *S. mitis* into the *S. mutans* biofilm, which also consisted of abundant extracellular matrix (figure 5 D).

Next, the same experimental setup was used to study the *S. mitis*/*A. actinomycetemcomitans* interactions. According to safranin-staining, *A. actinomycetemcomitans* was able to attach to the surface when *S. mitis* was inoculated as first bacterium, leading to monolayer formation (figure 4 C). The total mass was similar to that of *A. actinomycetemcomitans* mono-species culture (compare figure 3A). In the reverse seeding order, *A. actinomycetemcomitans* was enabled to form higher biofilm masses when seeded with CDM/sucrose compared to the mono-species culture. The inoculation of *S. mitis* led to a marginal decrease of biofilm mass compared to the mono-species control (*A. actinomycetemcomitans* + CS), but values were still higher as for the *A. actinomycetemcomitans* mono-species culture (figure 4 D).

Of note, viable cell counts for both bacteria decreased in this experiment, similar to the results obtained for the simultaneously seeded two-species culture (table S3). Fluorescence microscopic analysis of *A. actinomycetemcomitans* + CDM/sucrose and *A. actinomycetemcomitans + S. mitis* did not support the results obtained by safranin-stain. No obvious change in number of adherent cells and live/dead stain could be determined (data not shown).

### Transwell experiments

We next employed transwell assays to investigate if a change in biofilm mass and/or viable counts as seen in simultaneous and successive seeding experiments was caused by direct cell-cell contact or by soluble substances secreted by the tested species. Therefore, experiments were repeated in the above-mentioned combinations, but the bacteria were separated by a membrane with 0.2 µm sized pores.

**Figure 6:**
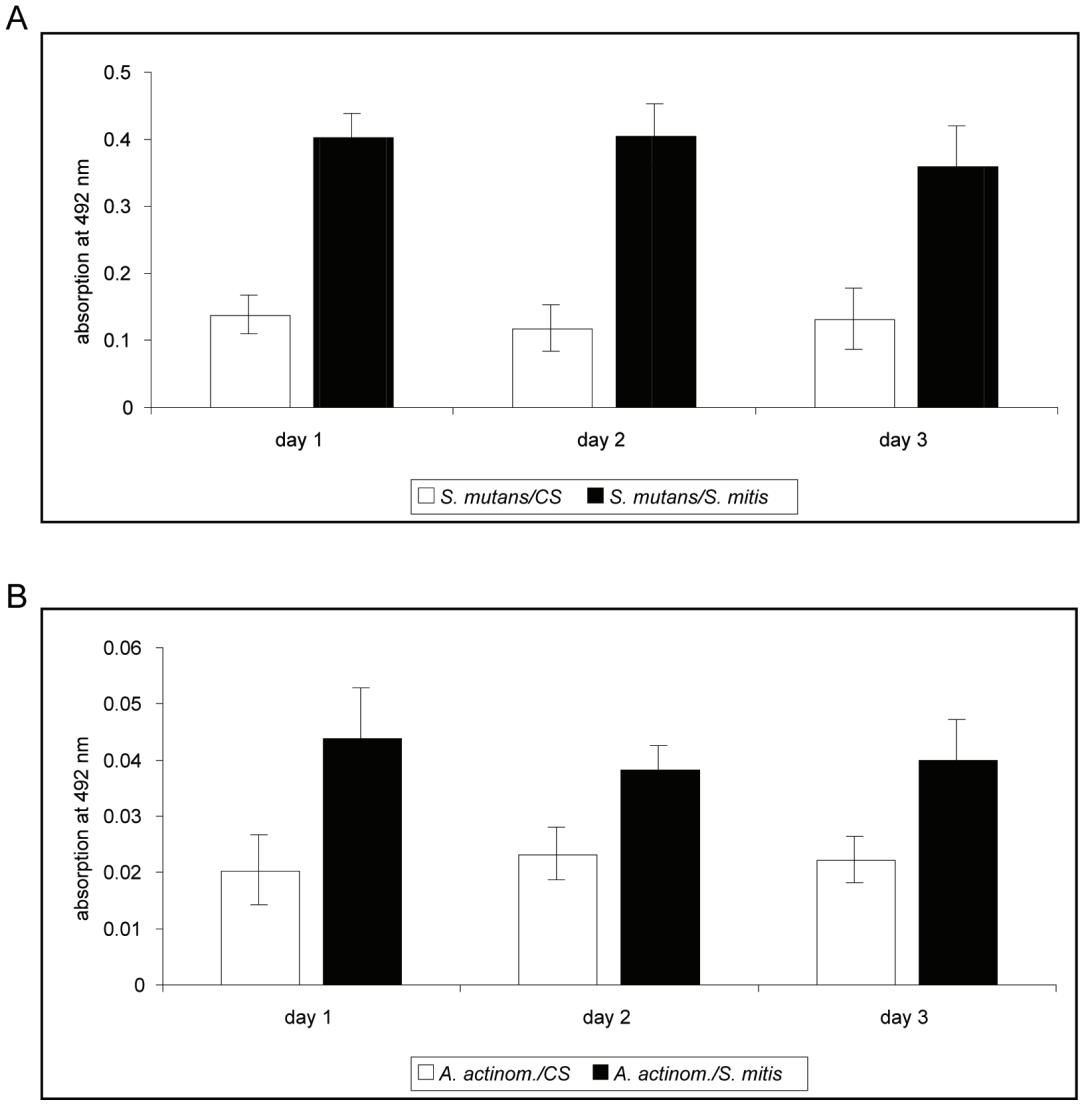
Results of safranin-staining assay after transwell experiments. *S. mitis* was inoculated in the upper compartment. A) and B) show the results for safranin-staining assays when *S. mutans* or *A. actinomycetemcomitans,* respectively, were inoculated in the lower compartment. For better optical discrimination, the grading of the y-axis is different in both graphs. CS – chemically defined medium with sucrose.

The data presented in figure 6 illustrate that *S. mutans* biofilm formation was stimulated in the presence of *S. mitis* (figure 6 A). Unfortunately, *A. actinomycetemcomitans* formed less dense mono-layers on the plastic of the transwell system compared to the 96-well polystyrene microtiter plates. However, adherence of this bacterium increased under the influence of *S. mitis* (figure 6 B).

The corresponding colony counts for the combinations investigated in the transwell setup are shown in the table S4. Viable counts of *S. mitis* adherent cells decreased when *S. mutans* was seeded in the upper compartment. In turn, growth and viable counts of *S. mutans* were slightly enhanced by the presence of *S. mitis* in the upper compartment.

**Figure 7:**
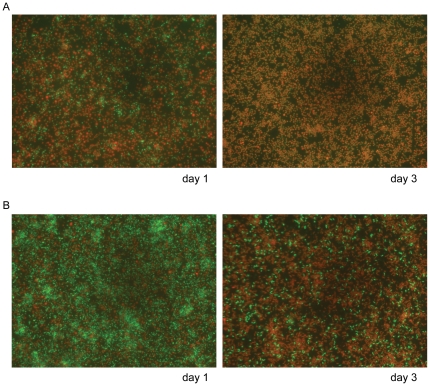
Fluorescence microscopy analysis of *A. actinomycetemcomitans* in the transwell system. A) *A. actinomycetemcomitans* mono-species culture in CDM/sucrose at days 1/3. B) *A. actinomycetemcomitans* grown in the remote presence of *S. mitis* on days 1/3. For the assay the cells were stained with the Live/Dead dyes. Live cells are stained in green, dead cells light up in red. Magnification: 400× for every picture.

Viable counts of *A. actinomycetemcomitans* were reduced to zero when *S. mitis* was present in the upper compartment, whereas *S. mitis* viability was marginally affected (table S4). In contrast to the results from the viable counts, rod shaped and, according to their green stain, viable *A. actinomycetemcomitans* cells were detectable in presence of *S. mitis*, when using fluorescence microscopy after live/dead staining. Moreover, viable *A. actinomycetemcomitans* cells were still present at day three of co-incubation with *S. mitis* (figure 7).

### Biofilm susceptibility to degrading substances

To analyze the nature of extracellular substances involved in formation of biofilm masses, we investigated the effect of pronase, DNase and sodium metaperiodate on established biofilms/monolayers. The increase or decrease of the biofilm mass subsequent to exposure would indicate prominent functions of proteins, extracellular DNA (eDNA) or carbohydrates, respectively, in biofilm mass and structure.

For *S. mutans* mono-species biofilms, an increase of biofilm mass could be observed when DNase was added to the 2 and 3 days old biofilm, whereas pronase treatment had such effects only on 2-day-old biofilms. The addition of sodiummetaperiodate led to a decreased biofilm mass of 1-day-old *S. mutans* biofilms, whereas treatment at days 2 or 3 caused no change. The analysis of *A. actinomycetemcomitans* monolayers with these substances revealed a significant decrease of safranin staining intensity when pronase was added to 1, 2 or 3 day old monolayers while the other substances had no obvious effects (figure S3 A–C).

For *S. mitis*, no biofilm formation was observed after the addition of pronase, DNase or sodiummetaperiodate (data not shown).

### Feasability of the basic setup for three-species communities

The next set of experiments was performed to prove the feasibility of the test system for three-species investigations. The mixture of *A. actinomycetemcomitans*, *S. mitis*, and *S. mutans* in a three-species culture revealed safranin values only marginally increased compared to the *S. mutans* mono-species culture (figure S4 A). In this combination only *S. mutans* was recovered from the established biofilm (figure S4 B). SEM and fluorescence microscopy visualized *S. mitis* and *S. mutans* bacteria in the biofilm, however, no *A. actinomycetemcomitans* was detected (figure S4 C and D).

## Discussion

The aims of this study were i) the establishment of an *in vitro* setup for mixed-species cultures from which the biofilm behavior of oral bacteria could be studied and which is easy and inexpensive to handle, as well as reproducible in other laboratories, ii) the introduction of a combination of complementary methods to substantiate results, and iii) the demonstration of its usefulness for investigating bacteria from the oral cavity of healthy subjects and/or patients with periodontitis (*S. mitis*, *S. mutans*, *A. actinomycetemcomitans*).

Initially, we sought the best suited medium for biofilm formation of the chosen bacterial species. Brain heart infusion medium (BHI) supplemented with human serum or saliva-like medium (SLM) was studied because of its similarity to sulcus fluid. Both media preparations hardly supported biofilm formation. The included human serum constituents [17] or mucin could potentially interfere with the bacterial adhesion to the plastic surface. Either a sterical hindrance after adhesion to the bacterial cell envelope or changes in the electrical charge of the bacterial or plastic surface are explanations of these effects.

Next, a chemically defined medium without glucose (CDM) supplemented with sucrose was tested and found to be optimal for the biofilm formation of *S. mutans, S. salivarius*, *S. sanguinis*, *S. intermedius* and the monolayer formation of *A. actinomycetemcomitans*. One major advantage of this medium is that its composition is known in detail, allowing an easy modulation of the presence and/or concentration of amino acids, phosphate or sugar. Unfortunately, the composition of CDM is more remote from sulcus fluid than that of the complex BHI medium. This potential disadvantage could not be resolved by the addition of human serum, due to the negative influence of the supplement on biofilm formation. Yet, a sucrose (or glucose) supplement has been established as an important substrate for the synthesis of extracellular polysaccharides, which in turn are crucial components of streptococcal biofilms [21], [22]. Therefore, the combination of CDM and sucrose was chosen for the experiments.

Although more closely reflecting the natural situation, fibronectin coating of surfaces did not significantly influence the biofilm forming ability of the tested mono-species as compared to uncoated supports.

Introduced washing steps in biofilm formation experiments are critically discussed [23]. For biofilms established under flow conditions Gomez-Suarez *et al.* [23] described detachment of bacteria from substratum surfaces after air-bubble exposure. However, under the chosen conditions in our study (static conditions) washing steps were crucial to remove sedimented bacteria.

When examining bacterial biofilms, several qualitative or quantitative measurements are established. Safranin-staining predominantly detects extracellular substances and is commonly used to quantify biofilm mass [24]–[26]. Viable cell counting identifies cells from biofilms which are able to multiply when transferred on fresh solid medium. Thus, both dead cells and viable but non-cultureable (VBNC) cells [27], [28] are not detected by this method although these cells contribute to total biofilm mass. By SEM, all cells can be visualized irrespective of their viability. Yet, due to the drying process, extracellular matrix is difficult to detect and visualize by this method. Fluorescence microscopy in combination with Live/Dead stain detects all cells, but not the extracellular substance. Multiplying and VBNC cells are simultaneously visualized as live cells. Finally, confocal laser scanning microscopy combined with Live/Dead stain principally detects the same objects as fluorescence microscopy, although the sterical assignment of cells allows to deduce the presence of extracellular matrix. Due to the different targets detected by the diverse methods, results obtained from a given biofilm could vary. The variation in turn allows conclusions about the association of cell numbers and amount of extracellular matrix, which could be produced by multiplying and VBNC cells. The present study demonstrates the necessity to examine bacterial biofilms with at least three different methods, i.e. safranin-staining, viable counts and microscopic inspection to obtain a complete picture.

Only by employing these three complementary methods, it became evident that *A. actinomycetemcomitans* and *S. mitis* behaved contrary in their planktonic growth and biofim behavior. Similar observations were previously reported by Fine and colleagues [29]. *A. actinomycetemcomitans* did not form multi-layered biofilms but covered the plastic surface by monolayers of viable but not multiplying cells. Several *A. actinomycetemcomitans* strains have been tested for biofilm formation with varying results [30]–[32]. Parameters like surface conditioning, growth medium and environmental conditions have been described to influence *A. actinomycetemcomitans* biofilm formation [33]. Obviously, different *A. actinomycetemcomitans* strains vary in their biofilm growth capabilities, with smooth colony formers growing to less biofilm mass and different biofilm structures [32], [34]. The present *A. actinomycetemcomitans* strain formed smooth colonies. The tendency of such strains to develop monolayers of viable cells for extended incubation periods has not been described so far. In general *A. actinomycetemcomitans* is known for its dependence on K^+^ ion concentration [35], slow growth rate, and limited carbon catabolic capabilities [36], which could possibly explain our observations with this species.

For *S. mitis* no biofilm formation could be observed under all tested conditions. Based on electron microscopy observations, Cowan et al. [37] demonstrated that *S. mitis* produced few, extremely long fibrils. These fibrils obviously enable the bacteria to adhere to the underlying substrate [38]. However, the cell surface of *S. mitis* differs from other oral streptococci in its content of nitrogen and oxygen rich polysaccharides [37]. This could be an explanation for the failure of *S. mitis* to form biofilm structures under the chosen conditions. The plastic surfaces of the used 96- and 24-well plates obviously did not support *S. mitis* adhesion. Previous studies demonstrated a dependence of *S. mitis* biofilm formation on the presence of acquired pellicle and lectins [39]. Similarly, the *S. oralis* strain C104 formed only small biofilm mass, leading to the conclusion that this species lacks effective colonization factors for binding to abiotic surfaces but can participate in complex biofilms by binding to more successful initial colonizers [40]. The latter statement is confirmed by the present observation on mixed *S. mitis/S. mutans* biofilms.

A notable result of this study is the obvious change in biofilm mass and viable counts in the two-species combinations compared to single-species settings. *S. mitis* has been described as a bacterium with an ecological control function in the oral cavity. Precisely, *S. mitis* could inhibit *A. actinomycetemcomitans* colonization [41]–[45]. Our results support this observation and associate the inhibitory effects of *S. mitis* to both the initial step as well as the ensuing multiplication ability of *A. actinomycetemcomitans*. In literature nutrient depletion and/or pH shift are discussed mechanisms for bacterial inhibitory effects [46]–[48]. However, if these mechanisms apply to the effects of *S. mitis* on *A. actinomycetemcomitans* is currently unknown.

Timing and spacing are two critical parameters in the development of mixed-species biofilms. Combinations of *S. mitis* with *S. mutans* always resulted in biofilm formation, although the final mass was determined by the timing of the bacterial adherence. Previous studies of van Hoogmoed et al. [42] uncovered the inhibition of *S. mutans* NS adhesion by biosurfactant-releasing *S. mitis* strains (*S. mitis* BA and *S. mitis* BM). These authors found a release of maximal amounts of biosurfactants, identified to be glycolipids, when the *S. mitis* strains were grown in the presence of sucrose. However, preliminary results from our laboratory indicate that this biosurfactant production could be a strain specific feature (data not shown). In order to introduce spacing as parameter in the line of investigation, transwell experiments were performed. These studies uncovered discrepancies between cfu values and counts of live/dead-stained cells, suggesting the adoption of a VBNC-status of *A. actinomycetemcomitans* in indirect contact to *S. mitis*. Furthermore, the experiments with *S. mutans* as well as *A. actinomycetemcomitans* in the remote presence of *S. mitis* suggested a control function for *S. mitis* under both conditions. At least for the remote effects, production of secreted substances is the most obvious explanation. The chemical nature of these substances needs to be determined. However, it is known from literature that production of detergents, toxic substances as hydrogen peroxide and bacteriocins or bacteriocin-like inhibitory substances are likely candidates for this effect [49], [50].

Analysis of *S. mutans* mono-species biofilms in the presence of protein-, DNA- or carbohydrate-degrading substances showed an unexpected effect, i.e. an incubation time-dependent increase of biofilm mass induced by DNase and pronase. Others have shown that presence of DNase during biofilm development leads to a significant disturbance of biofilm formation [51], [52]. These discordant observations are most likely due to different experimental setups. In summary, our results indicate that removal of eDNA after complete biofilm maturation has beneficial effects on a further biofilm mass increase. This observation could also explain the biofilm mass-inducing effects of *S. mitis* in transwell experiments, i.e. a secretion of enzymes with proteolytic or DNase activity.

In summary, the combined analysis of biofilm formation via safranin stain, determination of cfu and fluorescence microscopy after live/dead-stain allowed fast, unambiguous and reproducible results. Scanning electron- and confocal laser scanning microscopy complemented these results. The whole setup for mono-species cultures could be applied to two- and three-species combinations. This allowed first insights in interactions of chosen bacteria, precisely a mutual influence on biofilm formation and structure, as well as on different levels of viability.

## Materials and Methods

### Bacterial strains and culture conditions

The bacterial strains *Streptococcus mitis* ATCC 11843, *Streptococcus mutans* DSM 20523, *Streptococcus sanguinis* DSM 20567, *Fusobacterium nucleatum* DSMZ 25586, *Porphyromonas gingivalis* W83 ATCC BAA-308, *Parvimonas micra* ATCC 33270 and *Aggregatibacter actinomycetemcomitans* (*A. actinom*.) DSMZ 11123 were purchased from commercial providers (DSMZ, Braunschweig, Germany and ATCC, Manassas, USA). *Streptococcus intermedius* AC 3105 was obtained from the strain collection of the university Aachen, Germany. *S. salivarius* K12 was kindly provided by Dr. J. Tagg, New Zealand. Unless otherwise specified, all *streptococci* and *A. actinomycetemcomitans* were cultured in brain heart infusion medium (BHI; Oxoid) at 37°C under a 5% CO_2_ – 20% O_2_ atmosphere. *P. gingivalis* was cultivated in BHI supplemented with 5 µg/ml hemin and 50 mM galactose, and *F. nucleatum* and *P. micra* were cultured in BHI supplemented 0.25% glutamate. The latter three species were grown at 37°C under an anaerobic atmosphere (10% CO_2_ - 10% H_2_ – 80% N_2_).

### Culture media for biofilm studies

For the optimization of biofilm formation, brain heart infusion (BHI, Oxoid, Wesel, Germany) was supplemented with human serum (Sigma, Hamburg/Seelze, Germany) (4∶1) or a saliva-like medium (SLM, 0.1% Lab Lemco Powder, 0.2% yeast extract, 0.5% peptone, 0.25% mucine from porcine stomach, type III (Sigma), 6 mM NaCl, 2.7 mM KCl, 3.5 mM KH_2_PO_4_, 1.5 mM K_2_HPO_4_, 0.05% urea, pH 6.7) (1∶3). Alternatively, a chemically defined medium (CDM, [53]) was used without any glucose or supplemented with either human serum (4∶1), 50 mM glucose or 50 mM sucrose.

### General setup of biofilm cultures

Bacteria were grown in BHI to stationary phase, washed with phosphate buffered saline (PBS, pH 7.4), and adjusted to a strain specific OD_600_ to obtain 1×10∧8 cells ml∧-1. Subsequently, each bacterial suspension was diluted 10-fold in culture medium and inoculated in polystyrene 24-well-plates (Greiner Bio-One, Frickenhausen, Germany). The bacteria were cultivated alone to establish mono-species biofilms. Alternatively, *S. mutans* or *A. actinomycetemcomitans* were cultivated in combination with *S. mitis* resulting in two-species biofilms.

Biofilm-cultures were grown in an anaerobic incubator under an appropriate atmosphere (80% N_2_, 10% CO_2_, 10% H_2_) at 37°C for periods up to 5 days under static conditions (unless otherwise indicated). The atmosphere of the incubator was saturated with water vapor to prevent exsiccation of the cultures and was constantly exposed to a platinum catalyst to decrease the content of short-chained fatty acids in the atmosphere.

For comparison, planktonic growth of the bacteria in each medium was monitored by batch culture under anaerobic conditions and measuring absorbance at 600 nm.

### Biofilm mass and viable counts

For this type of assay, 96-well polystyrene microtiter plates (Greiner Bio-One, Frickenhausen, Germany) were employed. The plastic surfaces of the 96-well plates were either used uncoated or were coated with human fibronectin (Roche) at a concentration of 50 µg/ml∧-1 overnight at 4°C. Prior to the inoculation of the bacteria, fibronectin was removed and wells were washed and air-dried. After incubation of the bacterial cultures, liquid medium was removed and wells were washed gently with PBS in order to remove non-adherent sedimented cells.

For determination of biofilm mass, wells were stained with 0.1% safranin for 15 min, washed with PBS and air-dried. Biofilm mass was quantified in the air-dried wells by measuring the absorbance at 492 nm with a microplate reader (Tecan reader).

Viable cell numbers from biofilm bacteria were obtained by thorough scraping and washing of the wells with PBS. The resulting suspensions were serially diluted in PBS and plated in 100 µl aliquots on BHI-agar. Colony forming units (cfu) were counted after two days of incubation. The distinct colony morphology allowed for differentiation between the species.

### Biofilm structure documentation

Mono- or two-species biofilms were cultured in uncoated 24-well polystyrene cell culture plates (Greiner Bio-One, Frickenhausen, Germany), each well containing a sterile, uncoated 13-mm-diameter plastic microscope coverslip (Nunc, Wiesbaden, Germany). After one to five days of incubation under anaerobic conditions, biofilms were gently washed with PBS, stained with BacLight Live/Dead (Molecular Probes, Eugene, Oregon) and inspected by fluorescence microscopy (BX60 microscope, Olympus, Hamburg, Germany). Visible biofilms were documented with an attached digital camera (Leica, Solms, Germany).

In parallel experiments, samples were prepared for scanning electron microscopy (SEM) studies as follows: biofilms on the coverslips were fixed for 24 h in a solution containing 2.5% glutardialdehyde. The coverslips were washed with 0.1 M Na-acetate buffer (pH 7.3) and dehydrated in a graded series of ethanol. Subsequently, coverslips were subjected to critical point drying with CO_2_, sputter-coated with gold (thickness approx. 10 nm), and examined with a Zeiss DSM 960A electron microscope.

For confocal laser scanning microscopy (CLSM) studies, biofilms were grown in glass-bottom chamber slides (Nunc) and cultured for up to three days under anaerobic conditions. Following incubation, biofilms were gently washed with PBS and stained with BacLight Live/Dead (Molecular Probes, Eugene, Oregon). Preparations were inspected with a Zeiss inverted microscope attached to a Leica TCS SP2 AOBS laser scanning confocal imaging system with an Argon laser at 488-nm excitation wavelength and an Helium/Neon laser at 546-nm excitation wavelength. 3D images were obtained using the IMARIS x64 software.

### Transwell biofilm assay

For transwell studies, uncoated 24-transwell polystyrene cell culture plates (Corning) with one coverslip per well were inoculated with 600 µl (10∧7 cfu/ml) of the first bacterial species in the lower compartment and 200 µl (10∧7 cfu/ml) of the second bacterial species in the upper compartment (transwell inserts). Furthermore, uncoated 96 transwell polystyrene microtiter plates were used, containing 200 µl of the first/50 µl of the second bacterial species in the lower/upper compartment, respectively. After one to three days of incubation, transwell inserts and liquid medium were removed. The wells were gently washed with PBS, and biofilms were analyzed for biofilm mass, cell number, as well as biofilm structure using microscopic techniques (see above).

### Successive seeding assay

Medium suspensions with 10∧7 *S. mitis* were inoculated as first bacterial species in an uncoated 96- or 24-well polystyrene plate, the latter with one coverslip per well, and incubated for 24 h under anaerobic conditions at 37°C. Following this incubation time, the liquid medium (containing remaining planktonic bacteria) was removed and 10∧7 *S. mutans* or *A. actinomycetemcomitans* suspended in growth medium were inoculated as the second bacterial species into the wells. Subsequently, well plates were again incubated anaerobically at 37°C for up to two days. Biofilm formation was analyzed on a daily basis for two consecutive days by determination of biofilm mass via safranin stain, cell number by counting of cfu/ml and fluorescence microscopy after staining with BacLight Live/Dead (Molecular Probes, Eugene, Oregon). This assay was also performed in reverse order with *S. mitis* as the second bacterial species inoculated.

### Disorganization of biofilms

The disorganization of biofilm was performed as described by Inoue et al. [29] with minor modifications. Mono-species biofilms were cultured in uncoated 96-well polystyrene microtiter plates (Greiner Bio-One, Frickenhausen, Germany) for 1 to 3 days under anaerobic conditions. Following incubation time, liquid medium was removed and wells were washed gently with PBS. Subsequently, 200 µl of pronase (500 µg/ml), DNase (90 units), or sodiummetaperiodate (10 mM) diluted in PBS were added into the wells and the microtiter plates were incubated for further two hours at 37°C under anaerobic conditions. Finally, the liquid was removed and biofilm mass was quantified by safranin-stain (see above).

### Reproducibility and statistics

Each assay was performed in at least 3 wells at a given time (technical replicates) and was repeated on at least 3 independent occasions (biological replicates). Where appropriate, statistical parameters (mean, standard deviation of mean, p-Values) were determined employing the Windows Excel program and the Mann-Whitney U Test. P-values less than 0.05 were considered as significant.

## Supporting Information

Figure S1 Regular growth curves of *A. actinomycetemcomitans*, *S. mitis*, and *S. mutans* in CDM/sucrose. Growth was monitored by OD600 nm measurements in hourly intervals. One representative experiment of at least three replicates is shown.
(0.20 MB TIF)

Figure S2 Results of safranin-staining assay for the mono-species biofilms on a fibronectin-coated surface. The graph shows the result of safranin-staining assay for the tested mono-species on uncoated and fibronectin-coated surfaces. Fn - fibronectin.
(0.15 MB TIF)

Figure S3 Results of safranin-staining assay for the mono-species biofilm disorganization with pronase, DNase and sodiummetaperiodate. A), B) and C) Results for 500 µg/ml pronase, 90 units DNase, 10 mM sodiummetaperiodate, respectively. SMP - sodiummetaperiodate, * means significance of p<0.05 and ** means significance with p<0.01. PBS was used as control.
(0.38 MB TIF)

Figure S4 Results of Safranin-staining assay, number of colony froming units and microscopic analysis of the *S. mitis*/*S. mutans*/*A. actinomycetemcomitans* three-species combination. A) Safranin-staining assay of the mono- and three species-biofilms of *S. mitis*, *S. mutans* and *A. actinomycetemcomitans*. B) Number of colony forming units for the mono- and three-species cultures. Bacteria in brackets were the corresponding combination partner in the three-species culture. C–D) SEM and fluorescence microcopy of the *S. mitis*/*S. mutans*/*A. actinomycetemcomitans* combination. White arrow: *S. mitis*; Black arrow: *S. mutans*.
(4.10 MB TIF)

Table S1 Summarized results for planktonic and biofilm growth of the different species in the tested media.
(0.09 MB DOC)

Table S2 Number of colony forming units obtained for the mono- and two-species cultures in CDM/sucrose.
(0.03 MB DOC)

Table S3 Number of colony forming units for the successive seeding experiment.
(0.02 MB DOC)

Table S4 Number of colony forming units obtained in transwell experiments.
(0.02 MB DOC)

## References

[1] Mager DL, Ximenez-Fyvie LA, Haffajee AD, Socransky SS (2003). Distribution of selected bacterial species on intraoral surfaces.. J Clin Periodontol.

[2] Haffajee AD, Teles RP, Socransky SS (2006). The effect of periodontal therapy on the composition of the subgingival microbiota.. Periodontol 2000.

[3] Diaz PI, Chalmers NI, Rickard AH, Kong C, Milburn CL (2006). Molecular characterization of subject-specific oral microflora during initial colonization of enamel.. Appl Environ Microbiol.

[4] Dawes C (2008). Salivary flow patterns and the health of hard and soft oral tissues.. J Am Dent Assoc.

[5] Socransky SS, Haffajee AD; Cugini MA, Smith C, Kent RL (1998). Microbial complexes in subgingival plaque.. J Clin Periodontol.

[6] Slots J, Ting M (1999). *Actinobacillus actinomycetemcomitans* and *Porphyromonas gingivalis* in human periodontal disease: occurence and treatment.. Periodontol 2000.

[7] Van Winkelhoff AJ, Loos BG, van der Reijden WA, van der Velden U (2002). *Porphyromonas gingivalis*, *Bacteroides forsythus* and other putative periodontal pathogens in subjects with and without periodontal destruction.. J Clin Periodontol.

[8] Riep B, Edesi-Neuβ L, Claessen F, Skarabis H, Ehmke B (2009). Are putative periodontal pathogens reliable diagnostic markers?. J Clin Microbiol.

[9] Schlafer S, Riep B, Griffen AL, Petrich A, Hübner J (2010). *Filifactor alocis* – involvement in periodontal biofilms.. BMC Microbiol.

[10] Paster BJ, Boches SK, Galvin JL, Ericson RE, Lau CN (2001). Bacterial diversity in human subgingival plaque.. J Bacteriol.

[11] Stingu C-S, Eschrich K, Rodloff AC, Schaumann R, Jentsch H (2008). Periodontitis is associated with a loss of colonization by *Streptococcus sanguinis*.. J Med Microbiol.

[12] Sjollema J, van der Mei HC, Uyen HM, Busscher HJ (1990). Direct observations of cooperative effects in oral streptococcal adhesion to glass by analysis of the spatial arrangement of adhering bacteria.. FEMS Microbiol Lett.

[13] Larsen T, Fiehn NE (1995). Development of a flow method for susceptibility testing of oral biofilms in vitro.. APMIS.

[14] Bradshaw DJ, Marsh PD, Schilling KM, Cummins D (1996). A modified chemostat system to study the ecology of oral biofilms.. J Appl Bacteriol.

[15] Bowden GH (1999). Controlled environment model for accumulation of biofilms of oral bacteria.. Methods Enzymol.

[16] Guggenheim B, Giertsen E, Schüpbach P, Shapiro S (2001). Validation of an *in vitro* Biofilm Model of Supragingival Plaque.. J Dent Res.

[17] Palmer RJ, Kazmerzak K, Hansen MC, Kolenbrander PE (2001). Mutualism versus Independence: Strategies of Mixed-Species Oral Biofilms In Vitro Using Saliva as the Sole Nutrient Source.. Infect Immun.

[18] Foster JS, Palmer RJ, Kolenbrander PE (2003). Human oral cavity as a model for the study of genome-genome interactions.. Biol Bull.

[19] Hansen SK, Rainey PB, Haagensen JA, Molin S (2007). Evolution of species interactions in a biofilm community.. Nature.

[20] Periasamy S, Kolenbrander PE (2009). *Aggregatibacter actinomycetemcomitans* Builds Mutualistic Biofilm Communities with *Fusobacterium nucleatum* and *Veillonella* Species in Saliva.. Infect Immun.

[21] Bowen WH (2002). Do we need to be concerned about dental caries in the coming millennium?. Crit Rev Oral Biol Med.

[22] Paes Leme AF, Koo H, Bellato CM, Bedi G, Cury JA (2006). The role of sucrose in cariogenic dental biofilm formation – new insight.. J Dent Res.

[23] Gomez-Suarez C, Busscher HJ, van der Mei HC (2001). Analysis of bacterial detachment from substratum surfaces by the passage of air-liquid interfaces.. Appl Environ Microbiol.

[24] Christensen GD, Simpson WA, Bisno AL, Beachey EH (1982). Adherence of slime-producing strains of *Staphylococcus epidermidis* to smooth surfaces.. Infect Immun.

[25] Fessia SL, Griffin MJ (1991). A method for assaying biofilm capacity on polyurethane-coated slides.. Perit Dial Int.

[26] Lembke C, Podbielski A, Hidalgo-Grass C, Jonas L, Hanski E (2006). Characterization of biofilm formation by clinically relevant serotypes of group A streptococci.. Appl Environ Microbiol.

[27] Xu H-S, Roberts N, Singleton FL, Attwell RW, Grimes DJ (1982). Survival and viability of nonculturable *Escherichia coli* and *Vibrio cholerae* in the estuarine and marine environment.. Microb Ecol.

[28] Oliver JD (2005). The Viable but Nonculturable State in Bacteria.. J Microbiol.

[29] Fine DH, Furgang D, Kaplan J, Charlesworth J, Figurski DH (1999). Tenacious adhesion of Actinobacillus actinomycetemcomitans strain CU1000 to salivary-coated hydroxyapatite.. Arch Oral Biol.

[30] Inoue T, Shingaki R, Sogawa N, Sogawa CA, Asaumi J (2003). Biofilm Formation by a Fimbriae-Deficient Mutant of *Actinobacillus actinomycetemcomitans*.. Microbiol Immunol.

[31] Kaplan JB, Meyenhofer MF, Fine DH (2003). Biofilm Growth and Detachment of *Actinobacillus actinomycetemcomitans*.. J Bacteriol.

[32] Amarasinghe JJ, Scannapieco FA, Haase EM (2009). Transcriptional and translational analysis of biofilm determinants of *Aggregatibacter actinomycetemcomitans* in response to environmental perturbation.. Infect Immun.

[33] Haase EM, Bonstein T, Palmer RJ, Scannapieco FA (2006). Environmental influences on *Actinobacillus actinomycetemcomitans* biofilm formation.. Archives of Oral Biology.

[34] Fine DH, Furgang D, Schreiner HC, Goncharoff P, Charlesworth J, Ghazwan G, Fitzgerald-Bocarsly P, Figurski DH (1999). Phenotypic variation in Actinobacillus actinomycetemcomitans during laboratory growth: implications for virulence.. Microbiology.

[35] Ohta H, Onoue T, Fukui K (2001). Energy metabolsim of Actinobacillus actinomycetemcomitans during anaerobic and microaerobic growth in low- and high potassium contiuous culture.. Microbiology.

[36] Brown SA, Whiteley M (2007). A novel exclusion mechanism for carbon resource partitioning in *Aggregatibacter actinomycetemcomitans*.. J Bacteriol.

[37] Cowan MM, van der Mei HC, Rouxhet PG, Busscher HJ (1992). Physico-chemical and structural properties of the surfaces of *Peptostreptococcus micros* and *Streptococcus mitis* as compared to those of mutans streptococci, *Streptococcus sanguis* and *Streptococcus salivarius*.. J Gen Microbiol.

[38] Vadillo-Rodríguez V, Busscher HJ, Norde W, de Vries J, van der Mei HC (2004). Relations between macroscopic and microscopic adhesion of *Streptococcus mitis* strains to surfaces.. Microbiology.

[39] Oliveira MR, Napimoga MH, Cogo K, Goncalves RB, Macedo MLR, Freire MGM, Groppo FC (2007). Inhibition of bacterial adherence to saliva-coated through plant lectins.. J Oral Science.

[40] Loo CY, Corliss DA, Ganeshkumar N (2000). *Streptococcus gordonii* Biofilm Formation: Identification of Genes that Code for Biofilm Phenotypes.. J Bacteriol.

[41] Teughels W, Kinder Haake S, Sliepen I, Pauwels M, van Eldere J (2007). Bacteria Interfere with *A. actinomycetemcomitans* Colonization.. J Dent Res.

[42] Van Hoogmoed CG, van der Kuijl-Booij M, van der Mei HC, Busscher HJ (2000). Inhibition of *Streptococcus mutans* NS adhesion to glass with and without a salivary conditioning film by biosurfactant-releasing *Streptococcus mitis* strains.. Appl Environ Microbiol.

[43] Van Hoogmoed CG, Geertsema-Doornbusch GI, Teughels W, Quirynen M, Busscher HJ (2008). Reduction of periodontal pathogens adhesion by antagonistic strains.. Oral Microbial Immunol.

[44] Sliepen I, Hofkens J, Van Essche M, Quirynen M, Teughels W (2008). Aggregatibacter actinomycetemcomitans adhesion inhibited in a flow cell.. Oral Microbiol Immunol.

[45] Sliepen I, Van Essche M, Loozen G, Van Eldere J, Quirynen M, Teughels W (2009). Interference with Aggregatibacter actinomycetemcomitans: colonization of epithelial cells under hydrodynamic conditions.. Oral Microbiol Immunol.

[46] Talarico TL, Dobrogosz WJ (1989). Chemical characterization of an antimicrobial substance produced by *Lactobacillus reuteri.*. Antimicrob Agents Chemother.

[47] Sreenivasan PK, Meyer DH, Fives-Taylor PM (1993). Factors influencing the growth and viability of *Actinobacillus actinomycetemcomitans*.. Oral Microbiol Immunol.

[48] Leriche V, Carpentier B (2000). Limitation of adhesion and growth of *Listeria monocytogenes* on stainless steel surfaces by *Staphylococcus sciuri* biofilms.. J Appl Microbiol.

[49] Law DJ, Dajani AS (1978). Interactions between *Neisseria sicca* and viridin B, a bacteriocin produced by *Streptococcus mitis*.. Antimicrob Agents Chemother.

[50] García-Mendoza A, Liébana J, Castillo AM, de la Higuera A, Piédrola G (1993). Evaluation of the capacity of oral streptococci to produce hydrogen peroxide.. J Med Microbiol.

[51] Petersen FC, Tao L, Scheie AA (2005). DNA binding-uptake system: a link between cell-to-cell communication and biofilm formation.. J Bacteriol.

[52] Perry JA, Cvitkovitch DG, Levesque CM (2009). Cell death in *Streptococcus mutans* biofilms: a link between CSP and extracellular DNA.. FEMS Microbiol Lett.

[53] van de Rijn I, Kessler RE (1980). Growth characteristics of group A streptococci in a new chemically defined medium.. Infect Immun.

