# Supplementary figures and images for: Setup of an In Vitro Test System for Basic Studies on Biofilm Behavior of Mixed-Species Cultures with Dental and Periodontal Pathogens
Source: PLoS One. 2010 Oct 1;5(10):e13135. doi: 10.1371/journal.pone.0013135 (PMC2948514; doi:10.1371/journal.pone.0013135)

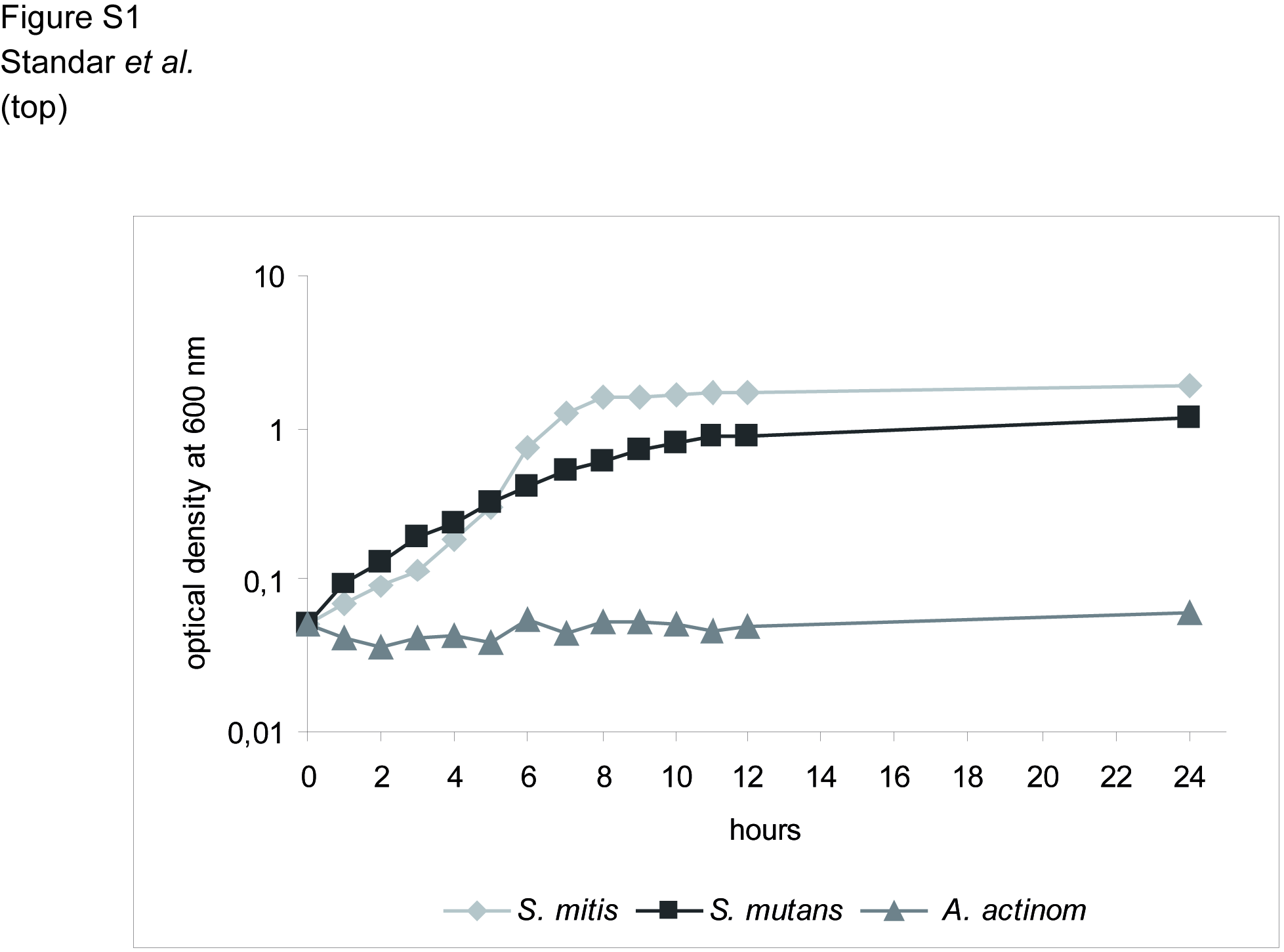

Supplement: Figure S1 — Regular growth curves of A. actinomycetemcomitans, S. mitis, and S. mutans in CDM/sucrose. Growth was monitored by OD600 nm measurements in hourly intervals. One representative experiment of at least three replicates is shown. (0.20 MB TIF) [file pone.0013135.s001.tif]

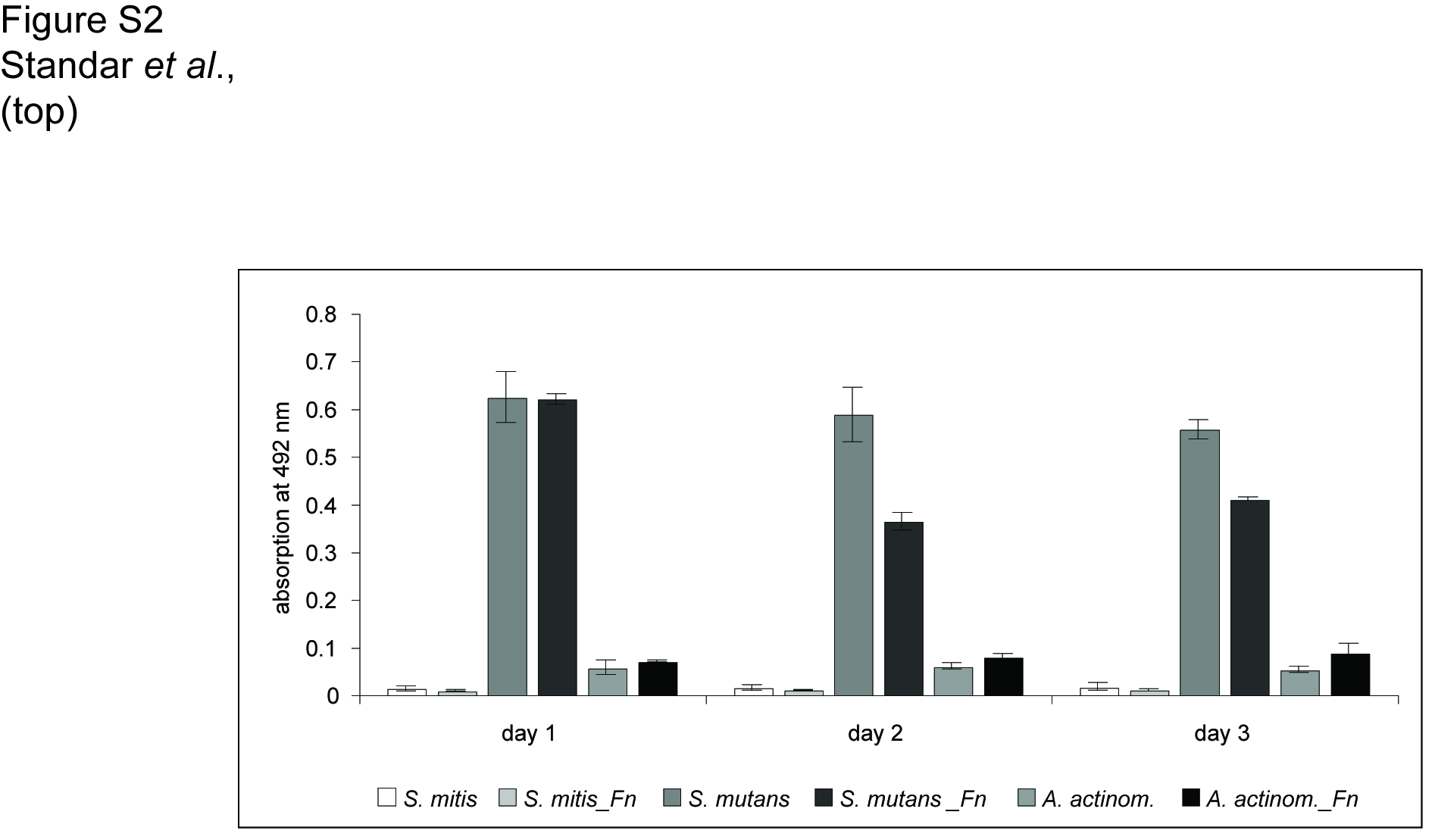

Supplement: Figure S2 — Results of safranin-staining assay for the mono-species biofilms on a fibronectin-coated surface. The graph shows the result of safranin-staining assay for the tested mono-species on uncoated and fibronectin-coated surfaces. Fn - fibronectin. (0.15 MB TIF) [file pone.0013135.s002.tif]

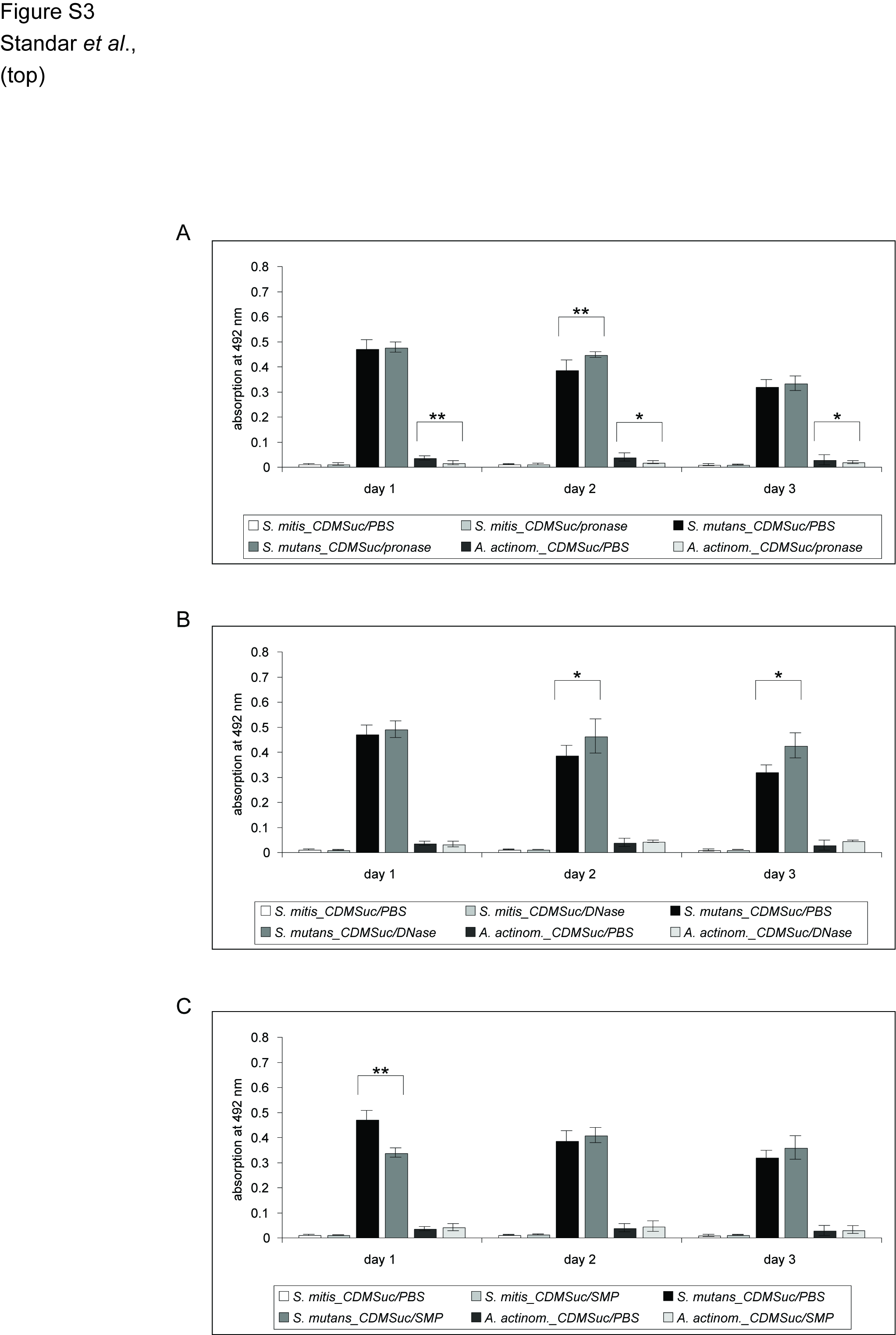

Supplement: Figure S3 — Results of safranin-staining assay for the mono-species biofilm disorganization with pronase, DNase and sodiummetaperiodate. A), B) and C) Results for 500 µg/ml pronase, 90 units DNase, 10 mM sodiummetaperiodate, respectively. SMP - sodiummetaperiodate, * means significance of p<0.05 and ** means significance with p<0.01. PBS was used as control. (0.38 MB TIF) [file pone.0013135.s003.tif]

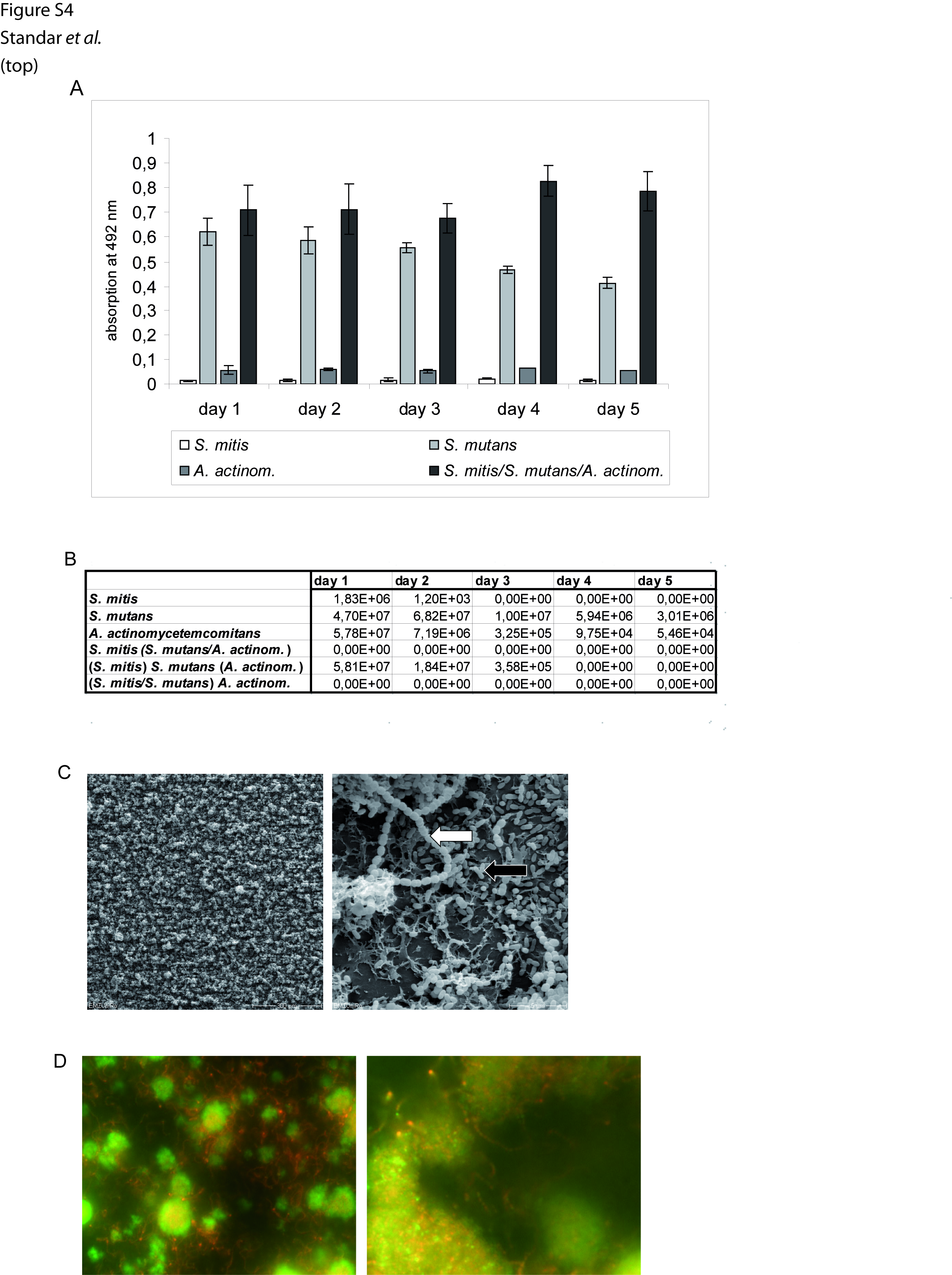

Supplement: Figure S4 — Results of Safranin-staining assay, number of colony froming units and microscopic analysis of the S. mitis/S. mutans/A. actinomycetemcomitans three-species combination. A) Safranin-staining assay of the mono- and three species-biofilms of S. mitis, S. mutans and A. actinomycetemcomitans. B) Number of colony forming units for the mono- and three-species cultures. Bacteria in brackets were the corresponding combination partner in the three-species culture. C–D) SEM and fluorescence microcopy of the S. mitis/S. mutans/A. actinomycetemcomitans combination. White arrow: S. mitis; Black arrow: S. mutans. (4.10 MB TIF) [file pone.0013135.s004.tif]
