# Supplementary material for: Setup of an In Vitro Test System for Basic Studies on Biofilm Behavior of Mixed-Species Cultures with Dental and Periodontal Pathogens
Source: PLoS One. 2010 Oct 1;5(10):e13135. doi: 10.1371/journal.pone.0013135 (PMC2948514; doi:10.1371/journal.pone.0013135)
Supplement: Table S1 — Summarized results for planktonic and biofilm growth of the different species in the tested media. (0.09 MB DOC) [file pone.0013135.s005.doc]

**Table S1: Summarized results for planktonic and biofilm growth of the different species in the tested media**

**A)**

B)

Table S1 A) presents the results for planktonic growth of the bacteria in the different media, monitored for batch cultures measuring the absorbance by 600 nm. For all cultures a starting OD600nm of 0.05 was chosen. Table S1 B) shows the results for biofilm growth in the tested media obtained by safranin-stainig. Plus/minus means biofilm formation/no biofilm formation, respectively. SLK – saliva like medium, HS – human serum, CDM – chemical defined medium, * indicates weak/few biofilm formation/growth.
