# Supplementary material for: Setup of an In Vitro Test System for Basic Studies on Biofilm Behavior of Mixed-Species Cultures with Dental and Periodontal Pathogens
Source: PLoS One. 2010 Oct 1;5(10):e13135. doi: 10.1371/journal.pone.0013135 (PMC2948514; doi:10.1371/journal.pone.0013135)
Supplement: Table S2 — Number of colony forming units obtained for the mono- and two-species cultures in CDM/sucrose. (0.03 MB DOC) [file pone.0013135.s006.doc]

**Table S2: Number of colony forming units obtained for the mono- and two-species cultures in CDM/sucrose.**

Bacteria in brackets were the corresponding combination partner in the two-species culture. Number of cfu/ml decreased over time for all tested bacteria. * no bacteria countable caused by an overgrowth of the combination partner.
