# Supplementary material for: Setup of an In Vitro Test System for Basic Studies on Biofilm Behavior of Mixed-Species Cultures with Dental and Periodontal Pathogens
Source: PLoS One. 2010 Oct 1;5(10):e13135. doi: 10.1371/journal.pone.0013135 (PMC2948514; doi:10.1371/journal.pone.0013135)
Supplement: Table S3 — Number of colony forming units for the successive seeding experiment. (0.02 MB DOC) [file pone.0013135.s007.doc]

**Table S3: Number of colony forming units for the successive seeding experiment.**

The first column designates the bacterial combinations used in these successive seeding experiments. The bacterial species on the left side in this column is the primary seeded bacterium; the species on the right side of this column is the subsequently seeded species. CDM/sucrose served as control.
