# Supplementary material for: Setup of an In Vitro Test System for Basic Studies on Biofilm Behavior of Mixed-Species Cultures with Dental and Periodontal Pathogens
Source: PLoS One. 2010 Oct 1;5(10):e13135. doi: 10.1371/journal.pone.0013135 (PMC2948514; doi:10.1371/journal.pone.0013135)
Supplement: Table S4 — Number of colony forming units obtained in transwell experiments. (0.02 MB DOC) [file pone.0013135.s008.doc]

**Table S4: Number of colony forming units obtained in transwell experiments.**

Bacteria or CS (CDMsucrose, used as control) in brackets mean their presence in the upper compartment of the transwell system.
